# Use of Synchrotron Phase-Sensitive Imaging for the Investigation of Magnetopriming and Solar UV-Exclusion Impact on Soybean (*Glycine max*) Leaves

**DOI:** 10.3390/cells10071725

**Published:** 2021-07-08

**Authors:** Anis Fatima, Sunita Kataria, Ashish Kumar Agrawal, Balwant Singh, Yogesh Kashyap, Meeta Jain, Marian Brestic, Suleyman I. Allakhverdiev, Anshu Rastogi

**Affiliations:** 1 Technical Physics Division, Bhabha Atomic Research Centre, Trombay, Mumbai 400085, India; ashishka@rrcat.gov.in (A.K.A.); balwants@rrcat.gov.in (B.S.); yskashyap@barc.gov.in (Y.K.); 2 School of Biochemistry, Devi Ahilya Vishwavidyalaya, Khandwa Road, Indore 452001, India; mjjainmeeta@gmail.com; 3 Department of Plant Physiology, Slovak University of Agriculture, A. Hlinku 2, 94976 Nitra, Slovakia; 4 K.A. Timiryazev Institute of Plant Physiology, Russian Academy of Sciences, Botanicheskaya St. 35, 127276 Moscow, Russia; suleyman.allakhverdiev@gmail.com; 5 Laboratory of Bioclimatology, Department of Ecology and Environmental Protection, Poznan University of Life Sciences, Piątkowska 94, 60-649 Poznan, Poland; anshu.rastogi@up.poznan.pl; 6 Faculty of Geo-Information Science and Earth Observation (ITC), University of Twente, 7500 AE Enschede, The Netherlands

**Keywords:** phase-sensitive imaging, magnetopriming, UV exclusion, leaf venation, leaf hydraulics

## Abstract

The combined response of exclusion of solar ultraviolet radiation (UV-A+B and UV-B) and static magnetic field (SMF) pre-treatment of 200 mT for 1 h were studied on soybean (*Glycine max*) leaves using synchrotron imaging. The seeds of soybean with and without SMF pre-treatment were sown in nursery bags kept in iron meshes where UV-A+B (280–400 nm) and UV-B (280–315 nm) from solar radiation were filtered through a polyester filters. Two controls were planned, one with polythene filter controls (FC)- which allows all the UV (280–400 nm); the other control had no filter used (open control-OC). Midrib regions of the intact third trifoliate leaves were imaged using the phase-contrast imaging technique at BL-4, Indus-2 synchrotron radiation source. The solar UV exclusion results suggest that ambient UV caused a reduction in leaf growth which ultimately reduced the photosynthesis in soybean seedlings, while SMF treatment caused enhancement of leaf growth along with photosynthesis even under the presence of ambient UV-B stress. The width of midrib and second-order veins, length of the second-order veins, leaf vein density, and the density of third-order veins obtained from the quantitative image analysis showed an enhancement in the leaves of plants that emerged from SMF pre-treated seeds as compared to untreated ones grown in open control and filter control conditions (in the presence of ambient UV stress). SMF pre-treated seeds along with UV-A+B and UV-B exclusion also showed significant enhancements in leaf parameters as compared to the UV excluded untreated leaves. Our results suggested that SMF-pretreatment of seeds diminishes the ambient UV-induced adverse effects on soybean.

## 1. Introduction

One of the non-ionizing parts of the electromagnetic spectrum of solar radiation is ultraviolet radiation. Ultraviolet (UV) radiations are further divided into three ranges: UV-A (315–400 nm), UV-B (280–315 nm), and UV-C (100–280 nm). The UV-C and major part of UV-B radiations are absorbed by the earth’s ozone layer [1]. Even if around 20% of UV-B is able to pass through the ozone layer and reach the earth’s surface, it may be harmful to biological systems due to its high energy content. Anthropogenic activities resulted in the reduction of the ozone layer, due to which the percentage of UV-B reaching the earth increased [2,3]. This further resulted in an increasing interest of scientists to understand how plants with a sessile nature react to this increased level of UV-B radiation [2,3,4,5,6]. The different responses of high UV-B radiation on plant structure, morphology, physiology, and genetics have been intensively studied previously [2,4,5,7] where UV-B radiations have been observed to adversely impact the cell membrane and caused changes in plant photosynthesis and enzyme activities [2,8].

Seed priming methods are the pre-treatment of seeds prior to sowing for the purpose of improving the physiological state of the seeds so that the seed germinates more efficiently [9,10]. There are several seed priming methods practiced in agronomy for increasing the seed germination, crop growth, and yield [10,11,12,13]. Static magnetic field (SMF) is a seed pre-treatment method based on the interactions of electromagnetic fields with seeds which act as bio-stimulators for the growth of seeds and plants [8,14,15,16]. The effect of SMF on plants has been extensively studied over the past few years as magnetic field pre-treatment may provide a non-chemical solution to the plants [16,17,18]. Some of the previous studies reported stimulatory effects of SMF treatment on crops including rice, maize, soybean, and sunflower [15,18,19,20,21], whereas the others reported slow development [22]. It is thus predicted that various plant species respond in different ways to varied frequencies and intensities of the magnetic field [23,24,25]. Plants showed reactions to magnetic fields based on the intensity, flux density, and exposure time [16,25,26]. The enhanced germination percentage improved plant growth, photosynthesis and yield were observed due to SMF pre-treatment of seeds as compared to the untreated seeds under non-stress as well as under abiotic stresses such as salt, water, UV-B, and arsenic toxicity [8,15,16,17,18,27,28,29]. The effect of magnetopriming on plants can be best understood in the framework of two mechanisms, namely the ion cyclotron-resonance (ICR) and the radical-pair models (RPM) [16,30]. The RPM is currently the only possible mechanism demonstrating the function of cryptochromes as a candidate for magneto-reception [16]. The experimental and theoretical studies provide evidence that the application of magnetic fields increases the average radical concentration, increases radical lifetime, and escalates the probability of radical reactions with cellular components [30]. The radical pair intermediates, triplet yields, and emission intensity that occur in Photosystem I and II of green plants can be modulated by an external magnetic field. The increased water uptake compared to untreated seeds is explained by the assumption that the magnetic field interacts with ionic currents in the cell membrane of the plant embryo [31]. In addition to these mechanisms, the interaction between environmental impacts such as ionizing radiation (ultraviolet-UV) and the magnetic field influence as a repair mechanism has also been reported previously in chick embryos [32].

Magnetic field treatment with low flux densities and the exclusion of solar UV radiation are the two parts of radiation biology that have positive stimulating effects on leaf growth, venation, and photosynthesis [8,18,29]. The network of leaf venation is composed of minor veins and a midrib (major conducting vein), which provides mechanical stability to the leaf structure. The venation network has the important function of transportation of water, nutrients, and carbon to different plant tissues [33,34,35]. The hydraulic system associated with plant leaf veins plays a key part in photosynthetic gas exchange and growth determination [36]. The width of midrib and minor veins, leaf vein density (LVD) (known as the vein length per leaf area), and the vein number density (which is the number of veins per leaf area) are all directly related to leaf hydraulic conductivity and photosynthesis [37,38,39]. Both magnetic treatment and exclusion of solar UV radiation change plant photosynthetic function which is related to the midrib of the leaf venation. The positive effects of solar UV exclusion and SMF on the leaf venation (midrib width) have been individually studied using synchrotron-based X-ray phase-contrast imaging [25,40]. However, there have been no reports on X-ray imaging of leaf venation to the combination of SMF pre-treatment of seeds and the exclusion of solar UV radiation.

The relationship of leaf venation and hence leaf hydraulics with photosynthesis is not yet explored completely. Advancements in non-destructive X-ray imaging techniques have overcome the limitations of manual sectioning and staining of leaves for imaging. So far, X-ray imaging studies for various parts of the plant have been reported [34]. X-ray radiography and micro-computed tomography (µCT) studies of intact plant parts with synchrotron radiation have contributed to the understanding of plant anatomical structures [37,41,42,43,44,45,46].

The phase-contrast imaging (PCI) technique relies on phase variations which occur when the X-ray wave front transmits through a sample [47,48,49,50]. The technique overcomes the limitations of conventional absorption-based techniques. It is well suited for imaging weakly absorbing samples like leaves in non-destructive ways [37]. In the present study, we have used the soybean (*Glycine max*) variety JS-335 an economically important crop to investigate the effects of exclusion of solar UV radiation in plants grown from the seeds pre-treated with SMF for 1 h. The aim of the present study was to determine the changes in the width of the midrib and minor veins, length of minor veins (2° and 3°) of leaves, and leaf vein density through high-resolution X-ray imaging and relate it to leaf growth, photosynthetic rate, and stomatal conductance.

## 2. Materials and Method

The soybean (*Glycine max* (L.) var. JS-335) seeds were procured from the Indian Institute of Soybean Research in Indore, India. The experiment was conducted under natural sunlight at the open terrace of the School of Biochemistry, in Devi Ahilya Vishwavidyalaya, Indore (22°44′ N, 75°50′ E), India. The experimental period was between October 2018 to December 2018. After moistening the SMF-pretreated (MT) and untreated (UT) soybean seeds were further mixed with recommended fungicides *viz*Bevistin and Diathane M at 2 gm kg^−1^ seeds and *Rhizobium* culture (provided by National Fertilizer limited, New-Delhi, India) at 3 g kg^−1^ seeds before sowing. The uniform shape and size of seeds were sown in plastic nursery bags of 34 × 34 cm. The nursery bags were filled with a mixture of soil, sand, and organic manure in a 2:2:1 ratio, and ten seeds of soybean were sown; three bags were prepared for each treatment. In each bag, six plants of uniform size were maintained after germination.

### 2.1. Magnetic Field Generation

An electromagnetic field generator (“AETec” Academy of Embedded Technology, Delhi, India) was used for the generation of magnetic field for seed pre-treatment, as previously described by Kataria et al. [51].

### 2.2. Magnetic Treatment

For the experiments, the seeds were exposed to SMF treatment of 200 mT for 1 h (MT) on the basis of our previous study on soybeans [25]. Through the Gauss meter, we can measure the magnetic field generated between the poles. The current in coils was regulated to obtain the exact magnetic field for the SMF pretreatment. At 50 mT, the variation in the applied field was observed to be 0.6% in the horizontal and 1.6% in the vertical direction, whereas, at 300 mT the variation decreased to 0.4% and 1.2% in both directions, respectively. A temperature of 25 ± 5 °C was maintained during seed exposure to SMF. The seeds from the same lot were kept under conditions without any influence of the magnetic field served as untreated (UT) seeds.

### 2.3. UV-A+B and UV-B Exclusion 

**Figure 1:**
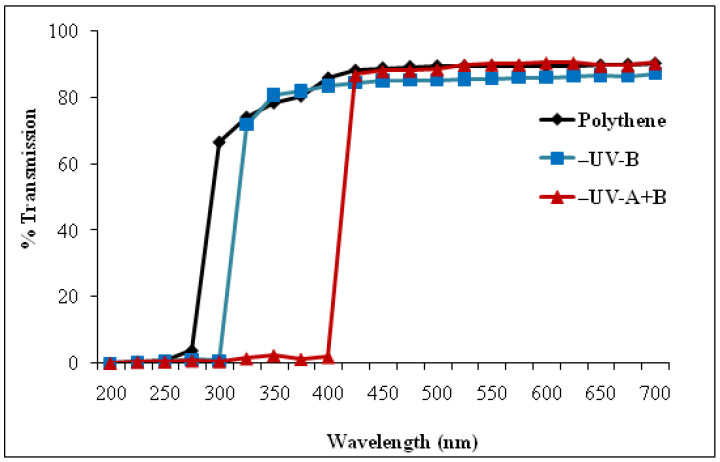
Transmission spectra of UV cut-off filters and polythene filter used for raising soybean plants under iron mesh cages [8] (Kataria et al. 2017a).

The UV-A+B and UV-B radiations were cut-off from solar radiation by using bandpass polyester filters (Garware polyester Ltd., Mumbai) with cut-offs of <315 nm and <400 nm radiation. Two controls were designed for this study; one with a polythene filter transparent to all ambient light (filter control, FC) and the other grown on the terrace without any filter (open control, OC). Figure 1 shows the transparency of the filters used in the experiments. The transmission spectra of the filters were measured according to the method of Kataria et al. [8]. The filters were continuously used from seed germination to maturity, with a regular exchange of filters every two weeks due to the solar radiation effect on the filters. For proper ventilation, the lower sides of the cage (0.35 m above the surface) holding the filter were not covered. The experiments were placed in the corner where sunlight was available throughout the day without any shading. The temperature inside and outside the cage was monitored through thermometers. During the growing period, average temperature was raised from 25 °C to 32 °C. No significant difference in the inside and outside temperatures was observed due to proper ventilation.

### 2.4. Radiation Measurement

At midday (around noon), a radiometer (Solar light Co. Inc. (PMA 2100), Glenside, PA, USA) was used to measure the intensity of solar spectra. The average photosynthetic active radiation (PAR) value at midday was observed to be 1450 µmol m^−2^ s^−1^ for the non-filter control, which decreased by 12.5% (1270 µmol m^−2^ s^−1^) under the UV-B filter and 11.8% (1280 µmol m^-2^ s^−1^) under the UV-A+B filter, whereas a decrease of 4.2% (1390 µmol m^−2^ s^−1^) was observed for the filter control.

### 2.5. Growth Data Collection and Analysis

A random selection of plants was done after 45 days of seed germination (DAE). At least three plants in triplicates from each treatment were harvested and transferred to the laboratory for growth data analysis. The soil particles from roots were washed and different parts of the plant were measured through a portable laser leaf area meter CID-202 scanning leaf area meter (CID Inc., Camas, WA, USA).

### 2.6. Photosynthesis and Stomatal Conductance 

The LI-COR photosynthetic system (Li-6200, LI-COR Inc., Lincoln, NE, Serial No. PPS 1332 USA) was used to measure net photosynthesis (*Pn*, μmol CO_2_ m^−2^ s^−1^) and stomatal conductance (*gs*, mol H_2_O m^−2^ s^−1^) for intact soybean plants from each experimental condition after 45 DAE. Photosynthetic measurements were performed on fully expanded third trifoliate leaves of soybean plants under ambient temperature and CO_2_ concentration, on clear days. The photosynthetic photon flux density (PPFD) was observed to be in between 1300–1600 μmol m^−2^ s^−1^ with airflow of 500 μmol s^−1^ and CO_2_ concentration of 350–380 ppm.

### 2.7. Phase Contrast Imaging Technique

The Imaging Beamline (BL-4), Indus-2 synchrotron radiation source [40,52] was used to generate the phase-contrast images. The experimental setup was previously described in [25].

The third trifoliate leaves of soybeans from all the groups were pressed flat and dried for two days at room temperature. The whole leaflets of the third trifoliate leaves were mounted in a rectangular metallic frame and phase-contrast images were acquired for middle regions in each leaf. The high-resolution X-ray microscope with 1.8 μm resolution (20 μm thick YAG-Ce scintillator, 4× objective, and PCO-2000 CCD camera) was used for image acquisition at 12keV energy, with a sample to detector distance (SDD) 50mm and an exposure time of 5 min.

### 2.8. Leaf Midrib Width Quantification

From the synchrotron images of the middle leaflet of third trifoliate leaves of soybeans, the midrib width was quantified at six places in the direction perpendicular to the length at fixed intervals with ImageJ [53]. The average width of the midrib vein and the adjoining minor vein (2°) was obtained for all the leaflets in the third trifoliate and an average value for the leaf was then calculated [25,40].

### 2.9. Leaf Minor Vein Length and Leaf Vein Density Quantification

The length of the minor vein (2°) was obtained using a freehand line in Image J. To obtain the total length and number of the (3°) minor vein in the entire phase contrast image of 2048 × 2048 pixel size, the objectJ plugin was used (plant-image-analysis.org/software/object (accessed on 12 April 2019). In the phase-contrast images, the vascular region above the midrib was selected with the freehand selection tool in Image J, and the area was measured. Similarly, the area of the vascular region below the midrib was acquired. To find the vascular area in the whole image, the area of the two regions measured were combined. Leaf vein density (LVD) was found by dividing the total length of all 3° veins (marked with red) in the image with the total area of the image. The total number of 3° veins in the images was divided with the total area to calculate the vein number density using ObjectJ.

### 2.10. Statistical Analysis

All data are presented in triplicate (*n* = 3); from each replica five plants were randomly taken for each treatment. The statistical analysis was performed on Microsoft Excel and Prism 4 (GrafPad Software, La Jolla, CA, USA) software where mean and standard errors were calculated, and the analysis of variance (ANOVA) followed by post hoc Newman–Keuls Multiple Comparison Test was performed. ^###^ *p* < 0.001; ^##^ *p* < 0.01; ^#^ *p* < 0.05 denotes statistically significant differences between seedlings that emerged from untreated (UT) seeds of OC with seedlings that emerged from untreated (UT) seeds of different treatment conditions-FC, UV-B and UV-A+B cutoff filters. *** *p* < 0.001; ** *p* < 0.01; * *p* < 0.05 denotes statistically significant differences between seedlings that emerged from SMF-pretreated (MT) and untreated (UT) seeds under each treatment.

## 3. Results and Discussion

In the present study, the individual effects of the exclusion of solar UV-A+B, UV-B radiation, and SMF pre-treatment as well as their combination were investigated on the growth, photosynthesis, and development of soybean leaves. Individual and joint exclusion of solar UV-A+B, UV-B radiation, and SMF pre-treatment significantly enhanced all leaf growth parameters studied in the present study, but the extent of enhancement was greater when the plants pre-treated with SMF were grown under ambient UV stress (OC and FC conditions).

**Figure 2:**
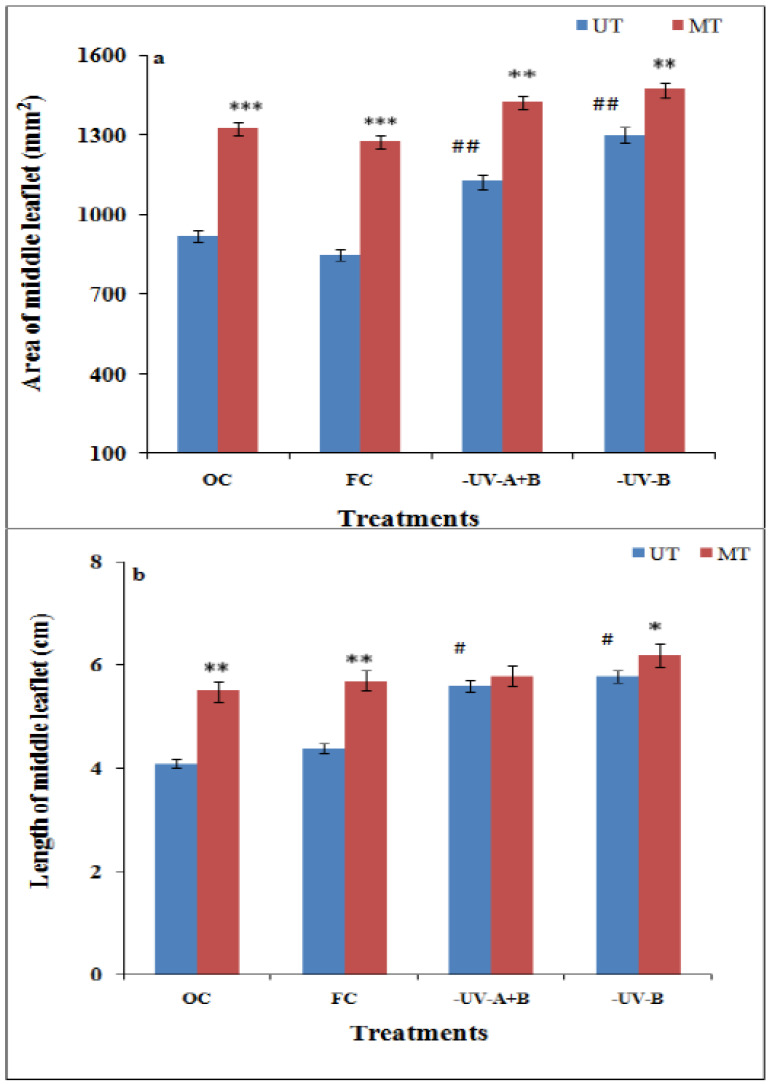

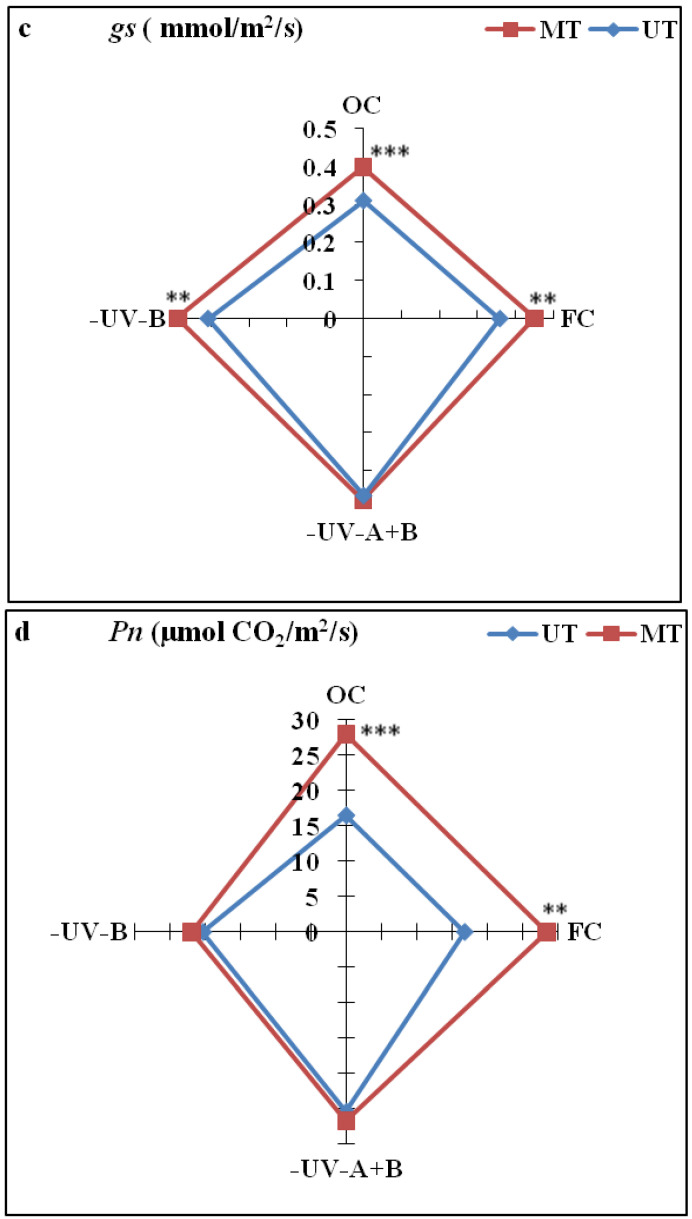
Leaf area (**a**), leaf length (**b**), stomatal conductance (**c**) and rate of photosynthesis (**d**) in middle leaflets of third trifoliate leaves of soybean after SMF pretreatment and solar UV exclusion in soybean. ^##^ *p* < 0.01; ^#^ *p* < 0.05 denotes statistically significant differences between seedlings emerged from untreated (UT) seeds of OC with the seedlings emerged from untreated (UT) seeds of different treatments conditions-FC, UV-B and UV-A+B cutoff filters, *** *p* < 0.001; ** *p* < 0.01; * *p* < 0.05 denotes statistically significant differences between seedlings emerged from SMF-pretreated (MT) and untreated (UT) seeds under each treatment.

A prominent increase was observed in the area and length of the middle leaflet of the third trifoliate leaves of soybean plants raised after SMF (200 mT for 1 h) priming with or without ambient UV radiations (Figure 2a,b). Similarly, solar UV exclusion also enhanced the area and length of middle leaflets of third trifoliate leaves of plants that emerged from untreated (UT) seeds (Figure 2a,b). The area of the middle leaflet increased by 44% and 50% through SMF-treatment respectively under OC and FC conditions as compared to their UT ones (Figure 2a).

The enhancement in the length of middle leaflets of third trifoliate leaves of soybean after SMF treatment was 34% in OC and 30% in FC conditions as compared to their UT ones (Figure 2b). A significant increase in leaf length by 41% under solar UV-B exclusion and 37% under UV-A+B exclusion in UT was observed as compared to the plants from UT seeds under OC conditions (Figure 2b).

A significant enhancement in stomatal conductance and photosynthetic rate was observed for the plants pretreated with SMF of 200 mT for 1 h (Figure 2c,d). SMF caused a 28% and 26% increase in stomatal conductance and a 70% and 69% increase in the net photosynthetic rate as compared with untreated controls respectively in OC and FC (presence of ambient UV stress) conditions (Figure 2c,d). Enhancement of leaf area along with an increase in the rate of photosynthesis and stomatal conductance after the SMF pre-treatment (200 mT for 1 h) has been previously reported in soybean and maize [8,15,18,21].

**Figure 3:**
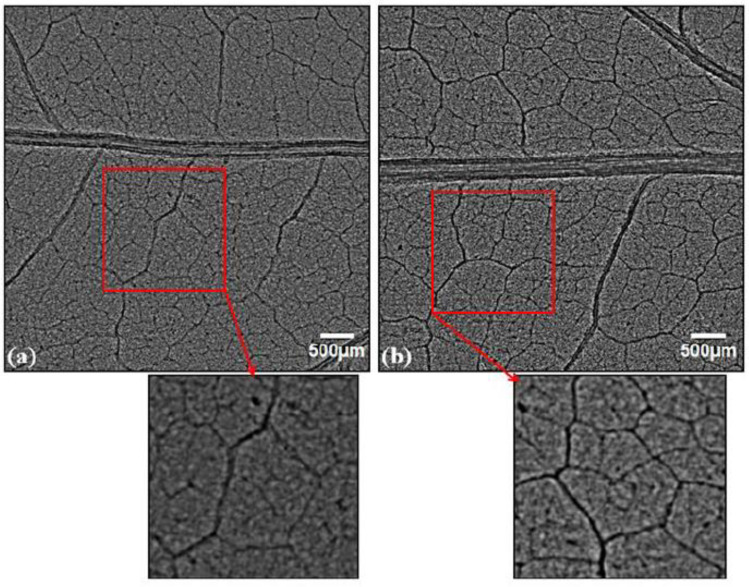
Phase contrast images of soybean leaves under open control (OC) receiving all ambient solar radiation: (**a**) emerging from untreated seeds, (**b**) emerging from seeds pre-treated with static magnetic field (SMF) of 200 mT strength for 1 h. The vascular region below the midrib region is highlighted in red and zoomed images are shown below the respective images.

**Figure 4:**
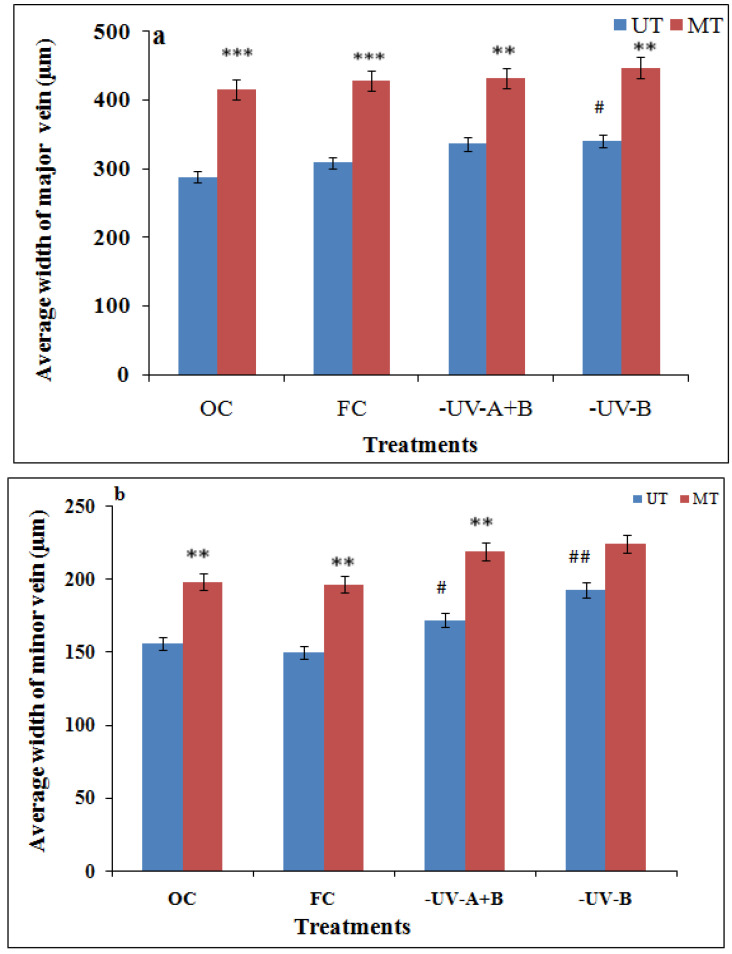

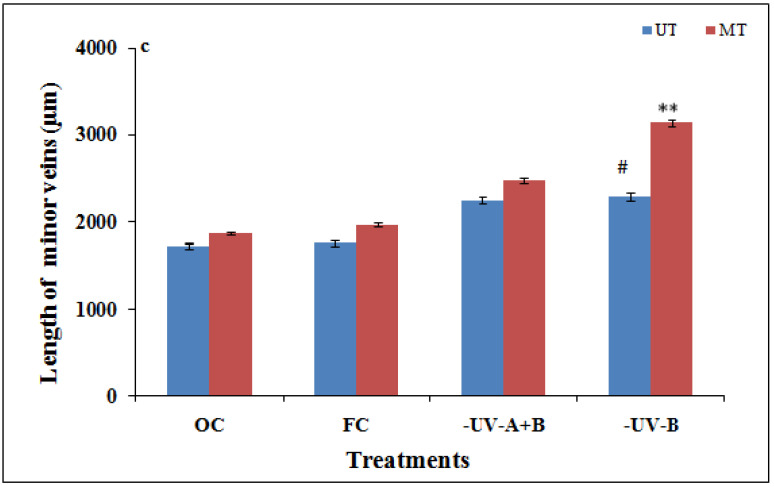
Width of midrib (**a**), width of minor veins (**b**) and length of minor veins (**c**) from X-ray images after SMF pretreatment and solar UV exclusion in middle leaflets of the third trifoliate leaves of soybean. ^##^ *p* < 0.01; ^#^ *p* < 0.05 denotes statistically significant differences between seedlings emerged from untreated (UT) seeds of OC with the seedlings emerged from untreated (UT) seeds of different treatments conditions-FC, UV-B and UV-A+B cutoff filters. *** *p* < 0.001; ** *p* < 0.01 denotes statistically significant differences between seedlings that have emerged from SMF-pretreated (MT) and untreated (UT) seeds under each treatment.

**Figure 5:**
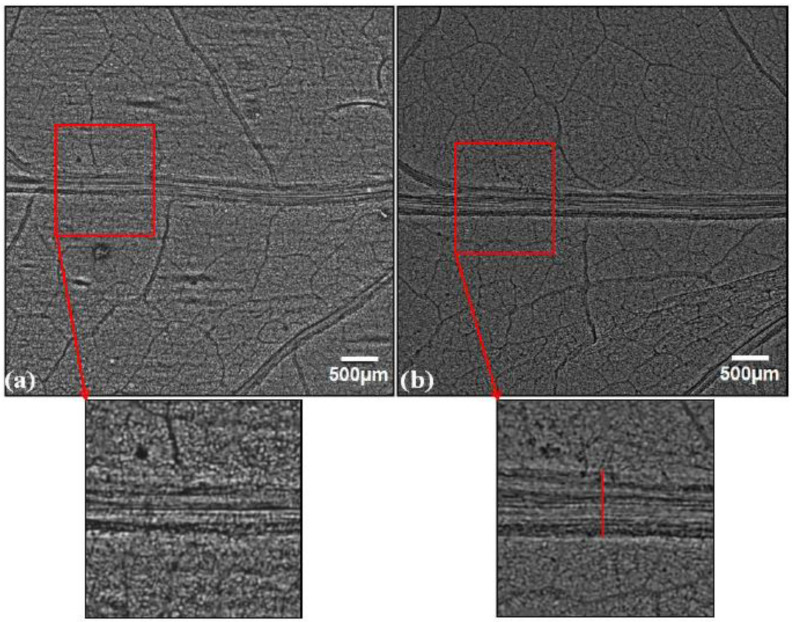
Phase contrast images of filter control (FC) soybean leaves grown with polythene filters which transmitted solar radiation: (**a**) emerging from untreated seeds, (**b**) emerging from seeds pre-treated with static magnetic field (SMF) of 200 mT strength for 1 h. The midrib regions enclosed with the red square in the images are zoomed to show midrib enhancement. The midrib quantification was done as shown with the vertical line in the zoomed filter control of the magnetically treated leaf (FCMT) image.

**Figure 6:**
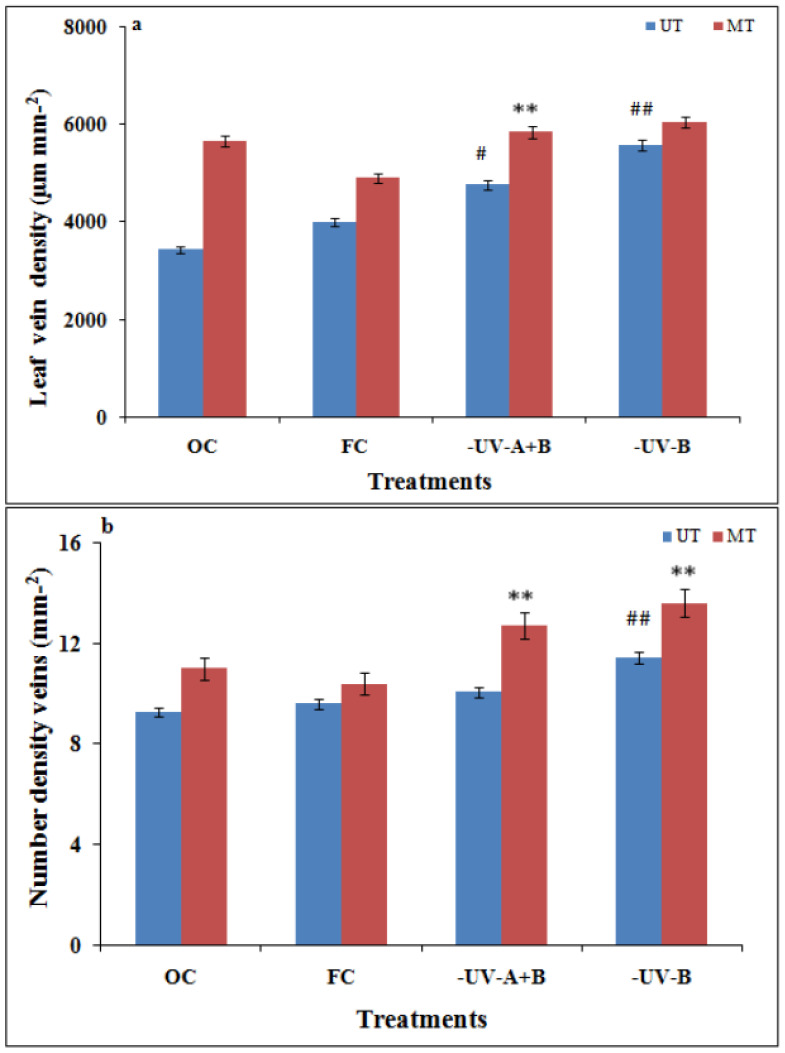
Leaf vein density of tertiary veins (**a**) and number density of veins (**b**) from X-ray images after SMF pretreatment and solar UV exclusion in soybeans. ^##^ *p* < 0.01; ^#^ *p* < 0.05 denotes statistically significant differences between seedlings that emerged from untreated (UT) seeds of OC with the seedlings that emerged from untreated (UT) seeds of different treatment conditions; FC, UV-B, and UV-A+B cutoff filters. ** *p*< 0.01 denotes statistically significant differences between seedlings that emerged from SMF-pretreated (MT) and untreated (UT) seeds under each treatment.

**Figure 7:**
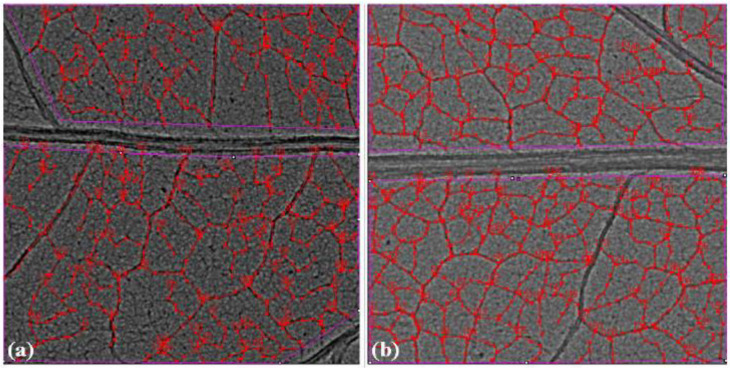
Phase-contrast images of soybean leaves from open control (OC) showing the 3° veins marked with red to obtain the total length of 3° veins and thus the leaf vein density (LVD) with the ObjectJ plugin. The number of minor veins in the images has been used to find the number density of the (3°) minor vein: (**a**) emerging from untreated seeds, (**b**) emerging from seeds pre-treated with a static magnetic field (SMF) of 200 mT strength for 1 h showing greater numbers of minor veins. Similar images for the quantification of 3° veins in other leaf groups have been obtained with ObjectJ.

**Figure 8:**
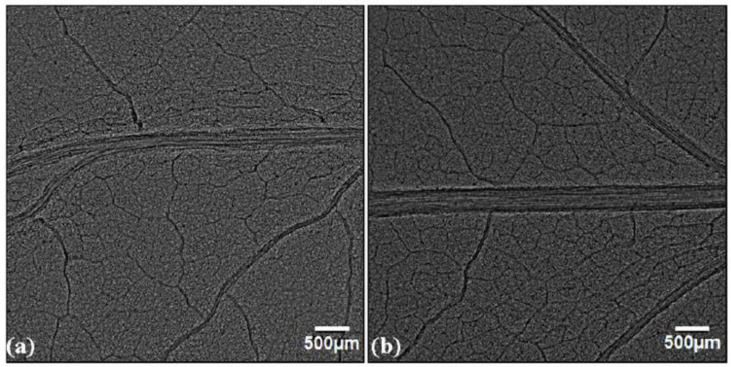
Phase contrast images of ultraviolet radiation excluded (UV-A+B) soybean leaves: (**a**) emerging from untreated seeds, (**b**) emerging from seeds pre-treated with static magnetic field (SMF) of 200 mT strength for 1 h.

**Figure 9:**
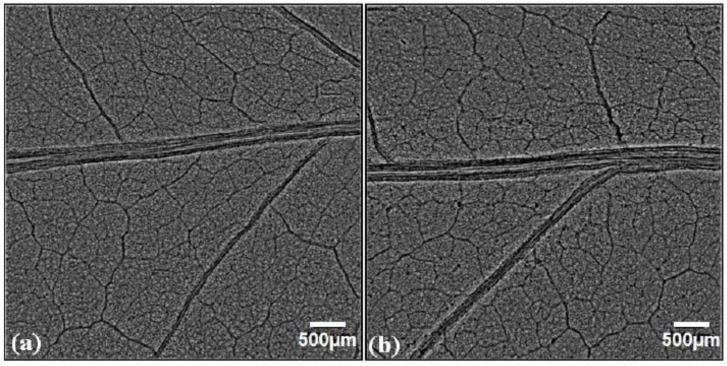
Phase contrast images of ultraviolet-B radiation excluded (UV-B) soybean leaves: (**a**) emerging from untreated seeds, (**b**) emerging from seeds pre-treated with static magnetic field (SMF) of 200 mT strength for 1 h.

A qualitative and quantitative comparison of phase-contrast images of untreated and SMF pre-treated leaves in OC, FC, UV-A+B, and UV-B showed enhancement in the midrib width, minor vein width, and leaf vascular region near the midrib (Figure 3, Figure 4, Figure 5, Figure 6, Figure 7, Figure 8 and Figure 9). In the OC group which received all the ambient solar radiation (280–400 nm), the quantification of leaf veins in the phase-contrast images showed an enhancement of 44% in the width of the midrib in the plants grown from the SMF pre-treated seeds as compared to untreated seeds (Figure 3 and Figure 4a). The visibility of vascular structures comprising of higher-order veins (3°) has also been improved in SMF pre-treated leaves (Figure 3b), which is due to a thinning effect [54].

The second-order (2°) minor veins also showed an increase of 27% in width and 8% in length by SMF treatment in the OC group (Figure 4b,c). Similar midrib enhancement in the SMF pre-treated group has been observed in the filter control leaves grown with polythene filters which received all the ambient solar radiation and also with UV cut-off filters (Figure 4a, Figure 5a,b, Figure 8 and Figure 9a,b). A 28% increase by UV-A+B and 31% by UV-B filters in the average width of major veins was observed after SMF treatment as compared to their UT ones (Figure 4a).

The zoomed images of the midrib region enclosed with rectangles in red (Figure 5a,b) show enhancement of the midrib structure in the SMF pre-treated leaves. Apart from the first- and second-order leaf veins, quantification of the tertiary veins (3°) has also been done with the ObjectJ plugin to obtain leaf vein density (LVD) (µm mm^−2^) and the number density of veins (mm^−2^) in leaves of all groups (Figure 6a,b). The tertiary veins, which are visible in the untreated and SMF pre-treated open control leaf images, are shown in red color (Figure 7a,b).

Comparison showed a higher LVD and a higher number of 3° veins in the SMF pre-treated group compared to the untreated group (Figure 6a,b and Figure 7a,b). In the OC and FC groups receiving all the solar radiation, SMF pre-treatment led to better growth of the plants, as observed from the synchrotron imaging results and also supported by the area and length of leaves and along with rate of photosynthesis in the plants. Thus, it indicated that SMF pre-treatment alleviated the UV stress in plants grown under OC and FC conditions receiving ambient solar radiation. 

In the UV-A+B and UV-B excluded group, the plants from untreated seeds (Figure 8a and Figure 9a) showed enhancement as compared to plants receiving ambient solar radiation (OC and FC) in terms of the width of the midrib and 2° vein, length of the 2° vein, LVD and number density of 3°veins (Figure 4 and Figure 6). The phase-contrast images for the combination of SMF pre-treatment and exclusion of solar UV-A+B and UV-B radiation (Figure 8b and Figure 9b) have also shown significant enhancement in the width of the midrib by 28% and 31% respectively, as compared to leaves that emerged from untreated seeds under UV exclusion filters (Figure 4a, Figure 8a and Figure 9a). The enhancement in the width of the midrib observed in UV-excluded along with SMF pre-treated leaves is lesser than the enhancements of 44% and 38% which were obtained in leaves of SMF pre-treated plants receiving all solar radiation respectively in OC and FC conditions (Figure 3, Figure 4a and Figure 5).

An increase in the leaf vein density and number density of minor (3°) veins was seen in the SMF pre-treated control leaves receiving all UV and also in the UV-A+B, UV-B excluded leaves (Figure 6a,b). Leaf vein density, which is the total length of minor veins per unit area, accounts for >80% of the total vein length [34]. The increase in the LVD of a minor (3°) veins indicates increased hydraulic activity in the SMF pre-treated leaves as reported [34].

High LVD can enable higher stomatal conductance and also indicates higher rates of gas exchange per unit leaf area and photosynthesis [25,39]. The vein density, leaf mid rib and minor vein thickness, were strongly correlated with the hydraulic conductivity and higher photosynthetic rate of the leaves. Thus, the observation showed that SMF pretreatment and solar UV exclusion individually and together enhanced leaf hydraulic efficiency, which can be observed through the changes in leaf venation architecture. The leaves were observed to be expanded with thicker veins from SMF-treated and UV excluded plants which give good mechanical support, whereas transpiration cooling and improved photosynthesis were observed because of higher water transportation due to higher vein length per unit area of the leaves [39,55]. The mechanism by which plants perceive MFs and regulate the signal transduction pathway is not fully understood. It has been suggested that MF perception/signaling in plants is regulated by blue light photoreceptors-cryptochromes. It has also been found that reactive oxygen species (ROS) and nitric oxide (NO) are the signaling molecules for magnetopriming-induced seed germination, plant growth, and photosynthesis [29,56]. The participation of NO through nitric oxide synthase enzyme was confirmed in SMF-induced tolerance towards UV-B stress in soybean [56]. However, this aspect of magneto biology still deserves in-depth investigation during leaf growth and photosynthesis.

## 4. Conclusions

The exclusion of UV-A+B and UV-B radiation is advantageous, as it was suggested that plant growth, leaf area, and photosynthesis were inhibited by ambient UV-B stress. The exposure of seeds to SMF treatment prior to sowing is an eco-friendly method with the potential to alleviate the adverse effects of UV-B stress in the plants. Looking into the correlation between leaf venation and leaf hydraulic conductivity, we used X-ray imaging to study leaf venation (major and minor vein up to 3°) under UV-exclusion, SMF pre-treatment, and the combined effect of both. UV exclusion and SMF pre-treatment individually and jointly showed positive effects on plant growth, development, photosynthesis, and leaf venation parameters obtained from the X-ray images. To our knowledge, this is the first study on X-ray imaging of leaf venation under the combined effects of solar UV exclusion and SMF pre-treatment.

## Data Availability

Users can obtain the datasets by being in touch with Anis Fatima (anees349@gmail.com), or Sunita Kataria (sunita_kataria@yahoo.com).
